# The Structures, Molecular Orbital Properties and Vibrational Spectra of the Homo- and Heterodimers of Sulphur Dioxide and Ozone. An Ab Initio Study

**DOI:** 10.3390/molecules26030626

**Published:** 2021-01-25

**Authors:** Thomas A. Ford

**Affiliations:** School of Chemistry and Physics, University of KwaZulu-Natal, Westville Campus, Private Bag X54001, Durban 4000, South Africa; ford@ukzn.ac.za

**Keywords:** ab initio, sulphur dioxide, ozone, dimers, complexes, structures, vibrational spectra

## Abstract

The structures of a number of dimers of sulphur dioxide and ozone were optimized by means of a series of ab initio calculations. The dimer species were classified as either genuine energy minima or transition states of first or higher order, and the most probable structures consistent with the experimental data were confirmed. The molecular orbitals engaged in the interactions resulting in adduct formation were identified and relations between the orbitals of the dimers of the valence isoelectronic monomer species were examined. The vibrational spectra of the most probable structures were computed and compared with those reported in the literature, particularly with spectra observed in cryogenic matrices. The calculations were extended to predict the properties of a number of possible heterodimers formed between sulphur dioxide and ozone.

## 1. Introduction

Among the family of non-covalent interactions [1], the chalcogen bond has enjoyed much prominence in recent years. The definition of a chalcogen bond is a “net attractive interaction between an electrophilic region associated with a chalcogen atom in a molecular entity and a nucleophilic region in another, or the same, molecular entity” [2]. Interest in non-covalent bonding in species containing oxygen and sulphur atoms has prompted us to revisit our theoretical study of the sulphur dioxide dimer [3], with particular emphasis on its vibrational spectrum, and to extend our computations to the dimer of the valence isoelectronic analogue ozone. The weakly-bound sulphur dioxide homodimer has been the subject of a number of theoretical investigations [3,4,5,6,7,8,9]; in most of these studies the authors considered a number of potential candidates for the global minimum structure, using a variety of medium-sized basis sets. Gas phase studies have been carried out on the dimer by microwave and radiofrequency spectroscopy [9,10,11,12], while the infrared spectrum of sulphur dioxide has been extensively investigated in cryogenic matrices [13,14,15,16,17,18,19,20,21,22,23]. In many of these vibrational studies the SO_2_ dimer has been specifically identified. Sulphur dioxide also forms binary complexes with a variety of atoms and other small molecules; theoretical studies have included those with H_2_S and HCN [5], H_2_O [5,11], Ar [6], BF_3_ [24], C_2_H_2_ [25], CHCl_3_ [26], NH_3_ and (CH_3_)_3_N [27], CO_2_, OCS, CS_2_ and N_2_O [28], NH_3_, H_2_O, HF, PH_3_, H_2_S and HCl [29], and CH_3_CN [30]. Many of these, and other, binary complexes have been observed in the gas phase, including those with H_2_O [11], BF_3_ [24], CH_3_CN [30], HF and HCN [31], C_2_H_2_ [32], C_5_H_5_N [33], (CH_3_)_2_O [34], CO_2_ [35], OCS [36], CS_2_ [37], N_2_O [38], and CHCl_3_ [39]. As was the case for the SO_2_ dimer, matrix isolation vibrational spectroscopy has also proved a fruitful source of data on binary complexes of sulphur dioxide. In this way, complexes of SO_2_ with CH_3_CN [30], Cl_2_, HBr, H_2_O, NH_3_ and C_2_H_4_ [40], NH_3_ and (CH_3_)_3_N [41], HF [42], H_2_O [43], C_6_H_6_ [44], and BF_3_ [45] in cryogenic matrices have been characterized.

Far less work has been reported on the analogous ozone dimer, indeed, only one theoretical study has apparently been carried out on this species [46]. The vibrational spectrum of ozone in cryogenic matrices has been investigated [16,47,48,49,50,51,52,53,54,55,56,57,58,59]. However, most of these studies have been more concerned with isotopic analysis [47,48,54,55], the geometry of the monomer [50], with fluorescence [53], or photochemical [55,56,57,58,59] reactions in the matrices. Only in one case was the ozone dimer mentioned [59], and in another example the appearance of additional absorptions was attributed to the existence of multiple trapping sites [51]. A number of binary complexes containing ozone have been observed in low-temperature matrices, including olefins [60], HBr, H_2_O, NH_3_, H_2_CO and C_2_H_4_ [61], PH_3_ [62,63], HF [42,64], H_2_O [65], H_2_O and SO_2_ [66], CH_2_F_2_ [67], CO [68], and O atoms [69]. In addition to the formation of binary complexes, ozone has been found to be a fairly reactive species in cryogenic matrices, and reactions between O_3_ and a range of small molecules and atoms have been analysed. These include reactions with CS_2_ and OCS [70], C_2_H_4_ [71], NO [72,73,74], NO_2_ [75], N_2_H_4_ [76], AsH_3_ [77], SbH_3_ [78], P_2_ and P_4_ [79,80,81], HCN [82], Cl_2_ and Br_2_ [83], Cl [84,85], and Ne atoms [86].

The SO_2_-O_3_ heterodimer has so far eluded investigation, either theoretically or experimentally.

## 2. Results and Discussion

### 2.1. Molecular Structures

**Figure 1:**
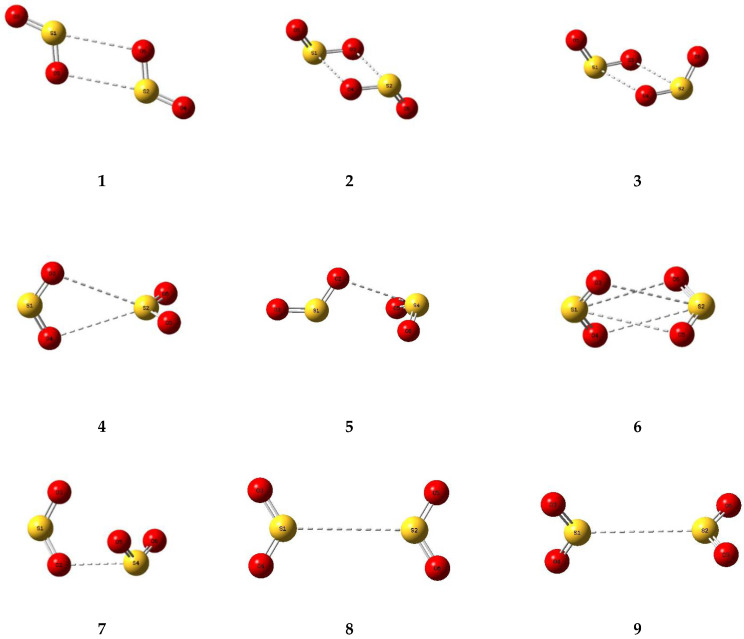
Optimized structures of some dimers of sulphur dioxide.

**Figure 2:**
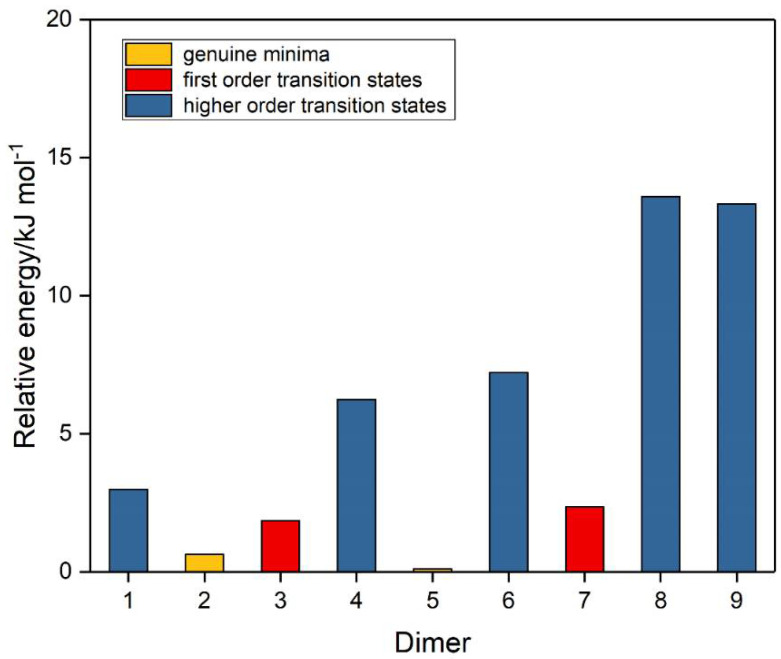
Relative energies of the sulphur dioxide dimers.

A number of trial structures were investigated for each adduct. We used as a template the publication of Hargittai [87], which examined a set of seven likely structural models for the metal dihalides, which we considered candidates as potential structures for the sulphur dioxide dimer. This trial set included three cyclic, two “linear”, and two bifurcated models, most of which were also investigated by other workers [3,4,5,6,8,9]. Along with the seven Hargittai structures, we also included two species that involved a S…S interaction, with no expectation that they would be strong candidates for the preferred SO_2_ dimer structure, but simply for completeness. Our nine possible dimer structures are illustrated in Figure 1 and their symmetries, energies, and Hessian indices are given in Table 1. Figure 2 shows their relative energies, separated according to their classification as genuine minima or transition states. We conclude, in agreement with the previous works [3,4,5,6,9] and with most of the experimental data [9,10,12,13,15,19,20,22,23], that our dimer **5** is the global minimum and is a non-symmetric species of C_s_ symmetry. The centrosymmetric dimer **2** is also a genuine minimum, being less than 1 kJ mol^−1^ higher in energy. Dimer **2**, however, being centrosymmetric, would not be observable by microwave spectroscopy, therefore, there is no possibility of identifying this structure in the gas phase. Table 2 reports the computed geometrical parameters of each dimer and their deviations from the corresponding monomer values. The perturbations of the bond lengths and bond angles from their monomer values are small (less than 0.3 pm and 0.6°, respectively), indicating a weak interaction in each case.

**Figure 3:**
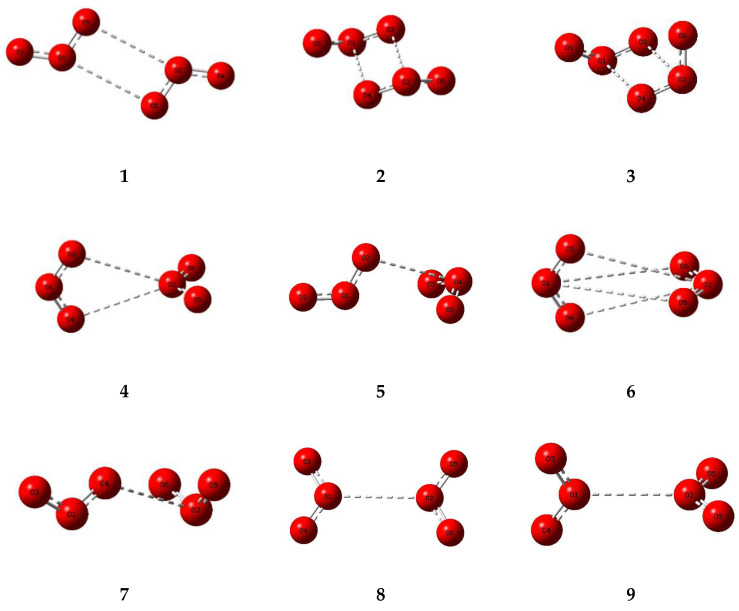
Optimized structures of some dimers of ozone.

**Figure 4:**
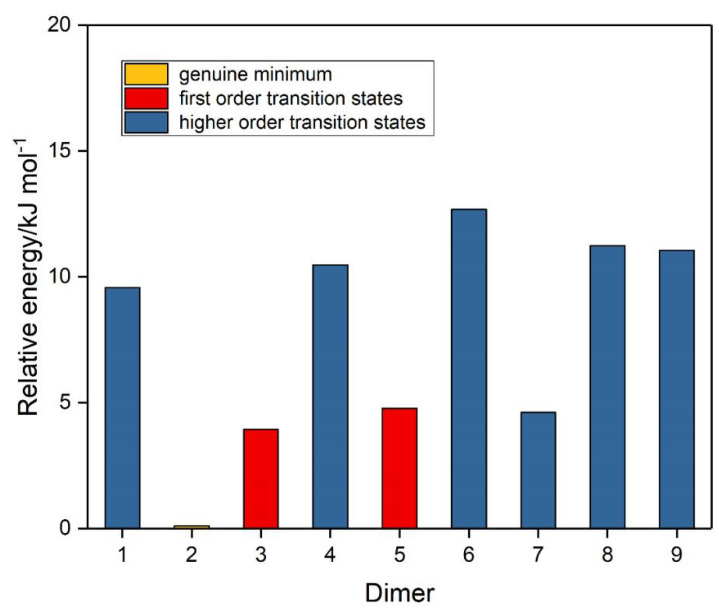
Relative energies of the ozone dimers.

The corresponding set of nine potential dimer structures for ozone are illustrated in Figure 3 and their properties are collected in Table 3. Their relative energies are presented in Figure 4. Only one of our ozone dimers (dimer **2**) was found to be a true minimum. This adduct corresponds with the second lowest energy sulphur dioxide dimer, while the counterpart of the C_s_ global minimum of (SO_2_)_2_ (ozone dimer **5**) is about 5 kJ mol^−1^ higher in energy. Our result is in contrast to that of Slanina and Adamowicz [46], who found the C_s_ counterpart of our dimer **5** to be the global minimum species. Part of the difference may be attributed to the use of different basis sets, but it has long been realized that the ozone monomer presents particularly formidable challenges for computation [88,89], and this is even more apparent for its dimer. The parameters of our ozone dimer **2** species are shown in Table 4. Again, the perturbations are minimal (less than 0.25 pm and 0.2°).

**Figure 5:**
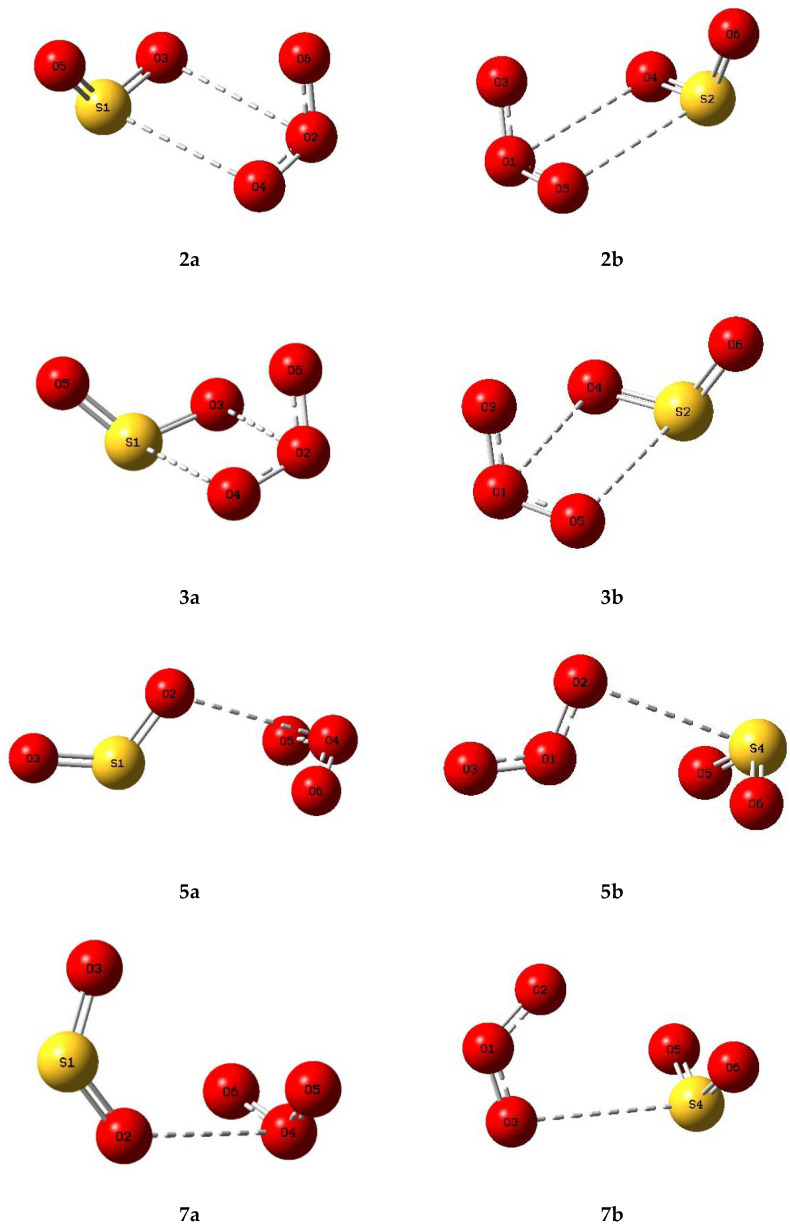
Optimized structures of some complexes of sulphur dioxide and ozone.

**Figure 6:**
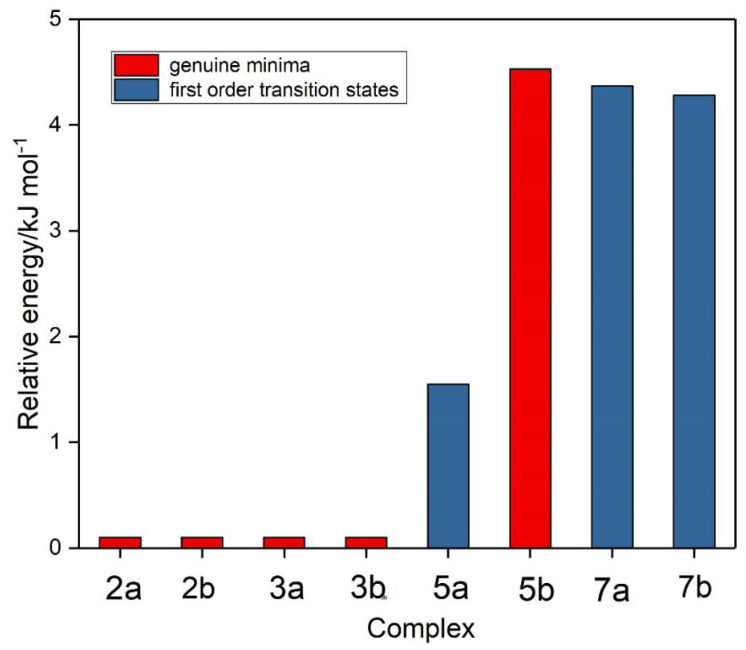
Relative energies of the sulphur dioxide-ozone complexes.

Based on the genuine minimum structures we found for the sulphur dioxide and ozone homodimers, and the C_s_ first order transition state of (O_3_)_2_, we examined eight structures for the sulphur dioxide-ozone heterodimer, two each corresponding with dimers **2**, **3**, **5**, and **7**, with sulphur dioxide and ozone acting as electron donor or electron acceptor in turn (a or b). These eight structures are illustrated in Figure 5 and their properties in Table 5. Structures **2a**, **2b**, **3a**, and **3b** were found to be virtually identical; these four structures and complex **5b** are all true minima. The relative energies are presented in Figure 6 and the bond lengths and angles and their changes in Table 6. Again, the pattern of very small perturbations is observed; only the free O2O6 bond of the ozone sub-unit in complex **2a** shows a substantial increase on complexation.

### 2.2. Interaction Energies

The interaction energies of the five genuine minimum energy structures are given in Table 7, corrected in each case for BSSE [90] and for zero-point energy differences. Consistent with the relatively insignificant intramolecular structural perturbations discussed above, the interaction energies are all less than 10 kJ mol^−1^; the two SO_2_ dimers and the **2a** heterodimer all have similar energies, while heterodimer **5b** is barely bound at all.

### 2.3. Molecular Orbital Properties

**Figure 7:**
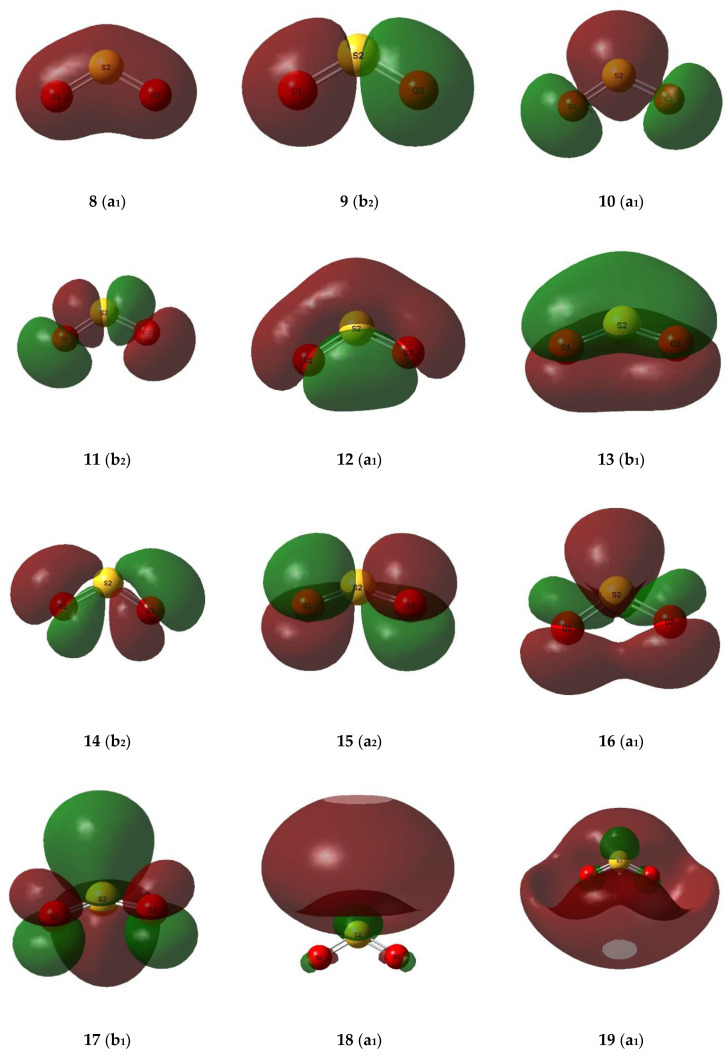
Valence molecular orbitals of the sulphur dioxide monomer.

**Figure 8:**
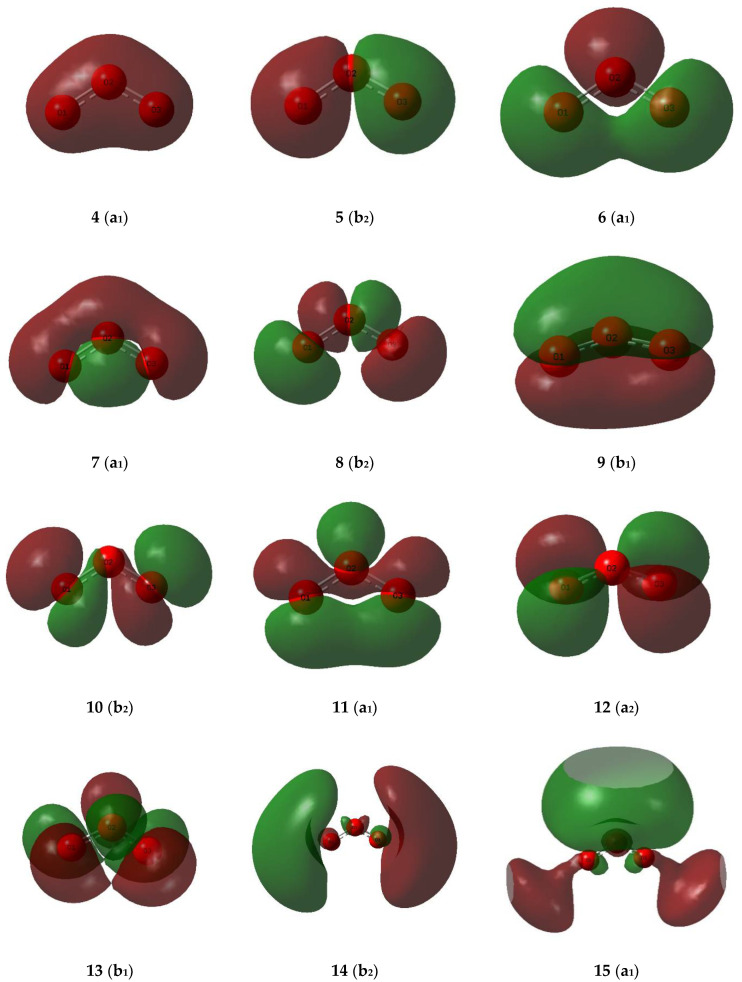
Valence molecular orbitals of the ozone monomer.

The valence molecular orbitals of the sulphur dioxide and ozone monomers are illustrated in Figure 7 and Figure 8, and their descriptions are listed in Table 8 and Table 9. The energy ordering of the orbitals follows the conventional sequence, σ < lp(O) ≈ lp(S) < π < π* < σ*. One sulphur and four oxygen lone pairs are expected for the SO_2_ monomer, and four terminal and one central oxygen lone pair in the case of O_3_. For both SO_2_ and O_3_ the π orbitals separate into a bonding orbital delocalized over all three atoms, a non-bonding orbital involving only the out-of-plane p orbitals of the peripheral oxygen atoms, and an antibonding orbital with contributions from the p orbitals of all three atoms. The σ* orbitals, being more diffuse and involving more excited atomic orbitals, are less easy to visualize and to assign.

These monomer orbitals transform readily into those of the dimer and complex species, and the orbitals of the five genuine minima are shown in the Supplementary Material as Figures S1–S5. The corresponding descriptions of the orbitals of the adducts are given in Supplementary Material Tables S1–S5. The major changes in the characters of the orbitals on complexation are that some of the lone pair orbitals of the monomers transform into σ bonding orbitals associated with the intermolecular bonding interactions. Thus, for SO_2_ dimers **5** and **2**, for example, four oxygen and one sulphur monomer lone pair orbitals go over into six oxygen and two sulphur lone pair orbitals, with two new σ(S…O) orbitals.

**Figure 9:**
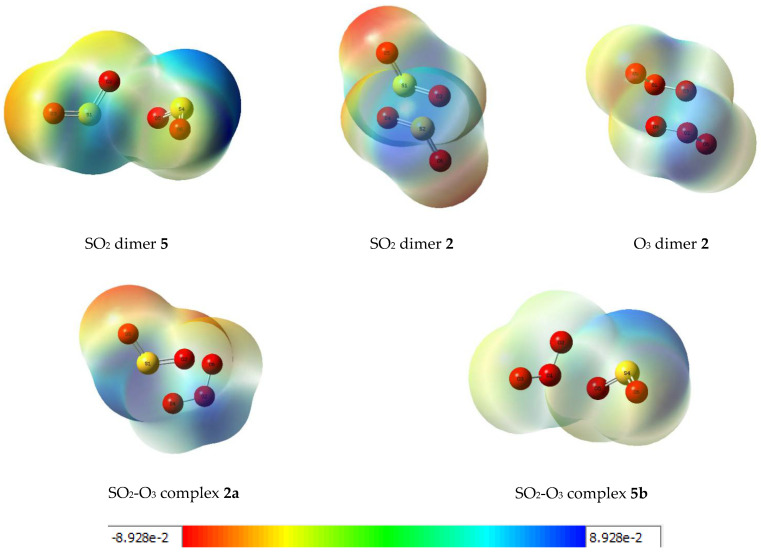
Molecular electrostatic potential plots of some dimers and complexes of sulphur dioxide and ozone. Units: hartree (1 *H* = 2625.346583 kJ mol^−1^).

Further insights into the electronic rearrangements accompanying dimer or complex formation are provided by a consideration of the molecular electrostatic potential maps of the adducts. These plots are shown in Figure 9 for the five associated species. The diagrams indicate the regions of high electron density, shown in red, shading to more electropositive zones, shown in blue, with the peripheral oxygen atoms having the greatest negative potentials and the more positive potentials associated with the sulphur atoms and the central oxygen atoms of the ozone moieties. The potentials cover a range from about −240 to 240 kJ mol^−1^.

### 2.4. Vibrational Spectra

The computed wavenumbers of the five associated species, and their shifts relative to the uncomplexed monomers, are shown in Table 10. For the two sulphur dioxide dimers, the antisymmetric SO_2_ stretching modes tend to be displaced to the red and the symmetric stretching and the SO_2_ bending to the blue. These shifts are all less than 10 cm^−1^ in either direction, however, consistent with the very low interaction energies (see Table 7). The comparisons of our calculated intramolecular wavenumbers with experimental values derived from matrix isolation infrared spectroscopic studies [13,15,19,20,21,22,23] are given in Table 11. As a measure of the level of agreement between the calculated and experimental wavenumbers of SO_2_ dimer **5**, the calculated/experimental ratios derived from the data in Table 11 are found to vary between 1.029 for ν_1_ (relative to ref. [23]) to 1.060 for ν_5_ (ref. [22]).

The vibrational data are all in agreement that the observed spectra are compatible with the C_s_ dimer **5** structure, except for the argon matrix results of Schriver-Mazzuoli et al. [21] and Ito and Hirabayashi [23], who proposed that the C_i_ isomer **2** more closely fits the experimental data. Indeed, Schriver-Mazzuoli and co-workers were able to assign only one band in each of the fundamental monomer regions with confidence, consistent with only one mode in each of the monomer regions being infrared-active [21]. There are some minor mismatches among the assignments of the stretching modes [20,23], but definitive assignments to the bands of the electron donor and acceptor based on the experimental spectra alone are difficult to achieve.

Table 10 includes the calculated data for ozone dimer **2**. The shifts of the antisymmetric O_3_ stretching modes are spectacularly large, given the low interaction energy of this dimer, and are of opposite sign. This result must be viewed in the context of the computed wavenumbers of the ozone monomer, 1157.9, 741.5, and 2244.3 cm^−1^ for ν_1_, ν_2_, and ν_3_, respectively, which may be compared with the experimental values of 1134.9, 716.0, and 1089.2 cm^−1^, reported by Barbe et al. [91]. This assignment admits a most unusual ordering of the stretching vibrations, with ν_1_ > ν_3_, which has been confirmed by Lee et al. [88,89]. Slanina and Adamowicz [46] report values of 1135, 726, and 2391 cm^−1^ for the monomer wavenumbers, in much closer agreement with our results. The antisymmetric stretching mode shifts indicate a significant separation of the two (in-phase and out-of-phase) vibrations of 247.3 cm^−1^, compared with separations of only 3.4 cm^−1^ and 1.7 cm^−1^ for SO_2_ dimers **5** and **2**, respectively, and of 17 cm^−1^ calculated for the C_s_ isomer of (O_3_)_2_ by Slanina and Adamowicz [46]. The anomalous position of the antisymmetric stretching wavenumber confirms the notoriously difficult task of accurately reproducing the experimental wavenumber of the ozone monomer theoretically [88,89,92,93]. These authors pointed out the multiconfigurational nature of the ground state of the ozone monomer, confirmed by a series of high-level computations, including at the CASSCF, MRCI, CCSD, and CCSD(T) levels of theory. In order to examine whether our treatment of the spectra of ozone and its dimer at the MP2/aug-cc-pVTZ level was sufficiently reliable, we repeated those calculations for the monomer at the CASSCF level. We selected the monomer for this test, since the experimental geometry [94] and vibrational spectrum [91] are well-established and are available for comparison. The comparisons of our geometrical parameters and vibrational wavenumbers at the MP2 and CASSCF levels with their experimental counterparts and with the parallel computations of Lee and Scuseria [89] are given in Table 12 and Table 13. While the computed bond length and angle are rather insensitive to the level of theory employed, and show fairly similar calculated and experimental differences (see Table 12), the estimation of the antisymmetric OO stretching mode exhibits a large scatter; only the theoretical treatment of Lee and Scuseria using CCSD/TZ+2Pf methodology [89] correctly reproduces the experimental ordering of the ν_1_ and ν_3_ vibrations (see Table 13). These uncertainties are responsible for the large, computed shifts associated with the monomer ν_3_ mode. Our shifts of the symmetric stretching and bending modes of the ozone dimer are much more in line with those of the SO_2_ dimers (less than 10 cm^−1^).

The computed wavenumber shifts of the SO_2_ moieties of SO_2_-O_3_ complexes **2a** and **5b** are quite consistent with those of the SO_2_ dimers (less than 5 cm^−1^, see Table 10). Similarly, the O_3_ shifts of heterodimer **5b** are insignificant, but those of the symmetric and antisymmetric O_3_ stretching modes of complex **2a** are quite substantial and of opposite sign, yielding a separation of 51.6 cm^−1^. While this separation is not as dramatic as the corresponding result for the C_i_ ozone dimer, it is quite apparent that the antisymmetric stretching vibrations of ozone molecules in these homo- and heterodimers are extremely sensitive to complexation.

## 3. Computational Methodology

The calculations were carried out using Gaussian-16 [95], at the second order level of the Møller–Plesset perturbation theory (MP2) [96] with Dunning’s augmented correlation-consistent polarized valence triple-zeta basis sets (aug-cc-pVTZ) [97,98]. Structures were optimized using the verytight keyword, where practicable, and stationary points were identified as genuine minima or transition states by vibrational analysis. The wavenumbers and infrared intensities of the resulting species were determined at the harmonic level. Interaction energies were computed and corrected for basis set superposition error (BSSE) [90], using the Boys–Bernardi full counterpoise procedure [99] and the counterpoise = 2 keyword and for zero-point energy differences. Molecular orbital properties and molecular electrostatic potentials were examined using the Gaussian input keywords pop = full, density = current, and cube(full,orbitals) [95]. The model chemistry employed here is consistent with those represented in a number of similar calculations [4,5,6,7,8] in terms of its ability to yield credible results.

## 4. Conclusions

A series of nine structures each of the sulphur dioxide and ozone homodimers and of eight of their heterodimers were investigated. Of these structures, two sulphur dioxide and one ozone dimer and two sulphur dioxide-ozone complexes were found to be genuine minima on their potential surfaces. These five species were all found to be very weakly bound (less than 10 kJ mol^−1^). These low interaction energies resulted in very small perturbations of the bond lengths and bond angles of the monomers (less than 0.36 and 1.39 pm for the SO and OO bond lengths and 0.29° and 0.58° for the OSO and OOO angles, respectively). The minimal perturbations of the intramolecular geometries are matched by the small, computed wavenumber shifts (less than 7 cm^−1^ for the SO_2_ dimers and the SO_2_ sub-units of the heterodimers, and less than 10 cm^−1^ for the symmetric stretching and bending of the O_3_ moieties of the ozone dimers and the heterodimers). The exceptions to this statement are ozone dimer 2, where the shifts of the antisymmetric O_3_ stretching mode are 175.0 and −72.3 cm^−1^, and SO_2_-O_3_ complex **2a**, where the O_3_ stretching vibrations undergo shifts of 25.8 cm^−1^ to the blue (antisymmetric) and the red (symmetric). The ozone moiety appears to be much more sensitive than sulphur dioxide to perturbations of their vibrational spectra due to complexation, but the conclusions regarding the magnitudes of the shifts have to be tempered by recognition of the well-known susceptibility of ozone to the level of theoretical treatment [88,89,92,93]. Miliordos and Xantheas presented evidence that, while the bonding in SO_2_ consists of two σ and two π bonds, that in O_3_ is better described as a mixture of a closed shell structure featuring two O-O bonds having bond orders of 1.5 (a delocalized 3-centre-4-electron bond) (82%) and a diradical structure with two σ bonds, a lone pair on the central oxygen atom, and a single electron in a p orbital on each of the terminal atoms (18%) [92]. Takeshita et al. concurred with respect to the description of the SO_2_ bonding arrangement (recoupled pair bonds), and the fact that O_3_ “has significantly more diradical character than SO_2_” [93]. These fundamental differences are certainly partly responsible for the marked variations in the ability of sulphur dioxide and ozone to form stable dimers, and hence in the differences in the computed vibrational spectra of the dimers. Neither Miliordos and Xantheas [92] nor Takeshita and co-workers [93] offered any insights into the spectra of the monomers, nor into the properties of the dimers.

We acknowledge the limitations of our methodology as they apply to the ozone species, but in the interests of consistency with our other results, we stand by the conclusions presented in this work.

## Figures and Tables

**Table 1:** Properties of some dimers of sulphur dioxide.

Dimer	Symmetry	E/*H*	Hessian Index	Relative Energy/ kJ mol^−1^
**5**	C_s_	−1095.9350 3678 41	0	0
**2**	C_i_	−1095.9347 9649 54	0	0.63
**3**	C_2_	−1095.9343 3272 07	1	1.85
**7**	C_s_	−1095.9341 4356 32	1	2.35
**1**	C_2h_	−1095.9339 0229 81	2	2.98
**4**	C_2v_	−1095.9326 6035 55	2	6.24
**6**	C_2v_	−1095.9322 8723 09	2	7.22
**9**	D_2d_	−1095.9300 0351 25	2	13.32
**8**	D_2h_	−1095.9298 6160 93	4	13.59

**Table 2:** Optimized geometrical parameters of sulphur dioxide dimers **5** and **2**, and their changes relative to the sulphur dioxide monomer. See Figure 1 for numbering of the atoms.

Dimer 5			Dimer 2		
Parameter	Dimer Value	Difference from Monomer Value	Parameter	Dimer Value	Difference from Monomer Value
r(S1O2)/pm	146.57	0.21	r(S1O3,S2O4)/pm	146.51	0.15
r(S1O3)/pm	146.32	−0.03	r(S1O5,S2O6)/pm	146.30	−0.05
r(S4O5,S4O6)/pm	146.41	0.06	∠O3S1O5,O4S2O6/deg	118.40	−0.39
∠O2S1O3/deg	118.21	−0.58	r(S1…O4,S2…O3)/pm	322.37	-
∠O5S4O6/deg	118.21	−0.58	∠O5S1…O4,O6S2…O3/deg	83.85	-
r(O2…S4)/pm	318.79	-	∠S1O3…S2,S2O4…S1/deg	106.60	-
∠S1O2…S4/deg	100.27	-	∠O5S1O3…S2/deg ^a^	73.23	-
∠O2…S4O5,O2…S4O6/deg	79.37	-	∠O6S2O4…S1/deg ^a^	−73.23	-
∠O5S4…O2S1/deg ^a^	−60.82	-			
∠O6S4…O2S1/deg ^a^	60.82	-			

^a^ Dihedral angle.

**Table 3:** Properties of some dimers of ozone.

Dimer	Symmetry	E/*H*	Hessian Index	Relative Energy/ kJ mol^−1^
**2**	C_i_	−450.2841 9179 490	0	0
**3**	C_2_	−450.2826 9146 051	1	3.94
**7**	C_s_	−450.2824 3399 654	2	4.61
**5**	C_s_	−450.2823 7307 965	1	4.77
**1**	C_2h_	−450.2805 4781 872	2	9.57
**4**	C_2v_	−450.2802 0490 636	4	10.47
**9**	D_2d_	−450.2799 8305 709	4	11.05
**8**	D_2h_	−450.2799 1140 084	5	11.24
**6**	C_2v_	−450.2793 6382 300	2	12.68

**Table 4:** Optimized geometrical parameters of ozone dimer **2** and their changes relative to the ozone monomer. See Figure 3 for numbering of the atoms.

Parameter	Dimer Value	Difference from Monomer Value
r(O1O3,O2O4)/pm	128.58	0.20
r(O1O5,O2O6)/pm	128.53	0.15
∠O3O1O5,O4O2O6/deg	116.49	−0.17
r(O1…O4,O2…O3)/pm	299.07	-
∠O5O1…O4,O6…O2O3/deg	65.08	-
∠O1O3…O2,O2O4…O1/deg	108.32	-
∠O5O1…O4O2/deg ^a^	−43.17	-
∠O6O2…O3O1/deg ^a^	43.17	-

^a^ Dihedral angle.

**Table 5:** Properties of some complexes of sulphur dioxide and ozone.

Complex	Symmetry	E/*H*	Hessian Index	Relative Energy/ kJ mol^−1^
**2a**	C_1_	−773.1098 0096 467	0	0
**3a**	C_1_	−773.1098 0095 885	0	0
**2b**	C_1_	−773.1098 0095 755	0	0
**3b**	C_1_	−773.1098 0095 165	0	0
**5a**	C_s_	−773.1092 1061 632	1	1.55
**7b**	C_s_	−773.1081 7161 056	1	4.28
**7a**	C_s_	−773.1081 3721 831	1	4.37
**5b**	C_s_	−773.1080 7683 409	0	4.53

**Table 6:** Optimized geometrical parameters of sulphur dioxide-ozone complexes **2a** and **5b**, and their changes relative to the sulphur dioxide and ozone monomers. See Figure 5 for numbering of the atoms.

Complex 2a			Complex 5b		
Parameter	Dimer Value	Difference from Monomer Value	Parameter	Dimer Value	Difference from Monomer Value
r(S1O3)/pm	146.72	0.36	r(O1O2)/pm	128.03	−0.35
r(S1O5)/pm	146.21	−0.15	r(O1O3)/pm	128.69	0.31
r(O2O4)/pm	127.85	−0.53	r(S4O5,S4O6)/pm	146.37	0.02
r(O2O6)/pm	129.76	1.39	∠O2O1O3/deg	116.50	−0.16
∠O3S1O5/deg	118.50	−0.29	∠O5S4O6/deg	118.51	−0.28
∠O4O2O6/deg	116.08	−0.58	r(O2…S4)/pm	313.42	-
r(S1…O4)/pm	291.71	-	∠O1O2…S4/deg	90.03	-
r(O2…O3)/pm	291.42	-	∠O2…S4O5,O2…S4O6/deg	83.45	-
∠O5S1…O4/deg	113.06	-	∠O5S4…O2O1/deg ^a^	59.89	-
∠O6O2…O3/deg	66.12	-	∠O6S4…O2O1/deg ^a^	−59.89	-
∠O5S1O3…O2/deg ^a^	107.94	-			
∠O6O2O4…S1/deg ^a^	55.56	-			

^a^ Dihedral angle.

**Table 7:** Interaction energies of some dimers and complexes of sulphur dioxide and ozone.

Species	Interaction Energy/kJ mol^−1^				
	Raw	BSSE	Corrected	ΔE_o_	Net
SO_2_ dimer **5**	13.26	2.38	10.88	1.93	8.95
SO_2_ dimer **2**	12.59	2.26	10.33	1.86	8.47
O_3_ dimer **2**	14.39	3.18	11.21	4.21	7.00
SO_2_-O_3_ complex **2a**	15.52	3.34	12.18	3.67	8.51
SO_2_-O_3_ complex **5b**	9.87	2.17	7.70	1.91	5.79

**Table 8:** Properties of the valence molecular orbitals of the sulphur dioxide monomer.

No.	Symmetry	Energy/*H*	Approximate Description ^a^
**1**–**7**			core
**8**	a_1_	−1.48513	σ(OSO)
**9**	b_2_	−1.38753	σ(OSO)
**10**	a_1_	−0.88026	lp(S)
**11**	b_2_	−0.69488	lp(O)
**12**	a_1_	−0.68513	lp(O)
**13**	b_1_	−0.65353	π(OSO)
**14**	b_2_	−0.54142	lp(O)
**15**	a_2_	−0.51405	π(nb)(OSO)
**16** (HOMO)	a_1_	−0.49779	lp(O)
**17** (LUMO)	b_1_	−0.00680	π*(OSO)
**18**	a_1_	0.06607	σ*(OSO)
**19**	a_1_	0.07129	σ*(OSO)

^a^ lp—lone pair; nb—non-bonding.

**Table 9:** Properties of the valence molecular orbitals of the ozone monomer.

No.	Symmetry	Energy/*H*	Approximate Description ^a,b^
**1**–**3**			core
**4**	a_1_	−1.74257	σ(OOO)
**5**	b_2_	−1.42739	σ(OOO)
**6**	a_1_	−1.09905	lp(O2)
**7**	a_1_	−0.82911	lp(O1) + lp(O3)
**8**	b_2_	−0.79824	lp(O1) − lp(O3)
**9**	b_1_	−0.77653	π(OOO)
**10**	b_2_	−0.56576	lp(O1) − lp(O3)
**11**	a_1_	−0.55546	lp(O1) + lp(O3)
**12** (HOMO)	a_2_	−0.48829	π(nb)(O1 − O3)
**13** (LUMO)	b_1_	−0.05229	π*(OOO)
**14**	b_2_	0.10144	σ*(OOO)
**15**	a_1_	0.10566	σ*(OOO)

^a^ O1 and O3—terminal atoms; O2—central atom. ^b^ lp—lone pair; nb—non-bonding.

**Table 10:** Wavenumbers and wavenumber shifts of some dimers and complexes of sulphur dioxide and ozone.

Species	SO_2_				O_3_			
	Symmetry	Mode ^a^	Wavenumber /cm^−1^	Shift /cm^−1^	Symmetry	Mode	Wavenumber /cm^−1^	Shift /cm^−1^
SO_2_ dimer **5**	a′	ν_1_ (ED)	1300.5	−5.0				
		ν_2_ (EA)	1102.5	3.3				
		ν_3_ (ED)	1099.1	−0.1				
		ν_4_ (OP)	500.0	6.7				
		ν_5_ (IP)	494.6	1.3				
	a″	ν_9_ (EA)	1303.9	−1.6				
SO_2_ dimer **2**	a_g_	ν_1_ (OP)	1303.2	−2.3				
		ν_2_ (IP)	1101.4	2.2				
		ν_3_ (IP)	494.9	1.6				
	a_u_	ν_7_ (IP)	1304.9	−0.6				
		ν_8_ (OP)	1101.5	2.3				
		ν_9_ (OP)	495.8	2.5				
O_3_ dimer **2**					a_g_	ν_1_ (OP)	2419.3	175.0
						ν_2_ (IP)	1150.2	−7.7
						ν_3_ (IP)	743.1	1.6
					a_u_	ν_7_ (IP)	2172.0	−72.3
						ν_8_ (OP)	1151.6	−6.3
						ν_9_ (OP)	742.8	1.3
SO_2_-O_3_ complex **2a**	a	ν_2_	1301.3	−4.2	a	ν_1_	2270.1	25.8
		ν_4_	1096.5	−2.7		ν_3_	1132.1	−25.8
		ν_6_	494.2	0.9		ν_5_	740.4	−1.1
SO_2_-O_3_ complex **5b**	a′	ν_3_	1102.4	3.2	a′	ν_1_	2243.8	−0.5
		ν_5_	495.3	2.0		ν_2_	1159.2	1.3
	a″	ν_9_	1306.2	0.7		ν_4_	744.7	3.2

^a^ ED—electron donor; EA—electron acceptor; IP—in-phase; OP—out-of-phase.

**Table 11:** Calculated and experimental intramolecular wavenumbers of sulphur dioxide dimer **5**.

Reference	Wavenumber/cm^−1 a^					
	ν_1_(a′) ν_a_(SO_2_)(ED)	ν_2_(a′) ν_s_(SO_2_)(EA)	ν_3_(a′) ν_s_(SO_2_)(ED)	ν_4_(a′) δ(SO_2_)(OP)	ν_5_(a′) δ(SO_2_)(IP)	ν_9_(a″) ν_a_(SO_2_)(EA)
This work ^b^	1300.5	1102.5	1099.1	500.0	494.6	1303.9
Ref. [13] ^c^	1343.1, 1345.2	-	-	-	-	1341.1
Ref. [15] ^d^	1348.2	1155.8	1153.7	524.0	521.7	1345.6
Ref. [19] ^d^	1349.2	1155.2	1153.9	526.1	524.0	1346.6
Ref. [20] ^d^	1346.6	1151.8	1154.2	524.3	522.4	1349.1
Ref. [21] ^c^	1341.3	1146.6	-	519.5	-	-
Ref. [22] ^c^	1345.1	1155.4	1153.3	527.2	521.8	1341.1
Ref. [22] ^d^	1349.4	1156.1	1154.3	526.5	524.3	1346.5
Ref. [23] ^c^	1345.8, 1346.5	1154.8, 1155.5	1152.1, 1152.8	-	-	1348.2, 1348.9
Ref. [23] ^e^	1344.4, 1345.2	1152.9, 1153.6	1149.9, 1150.5	-	-	1346.3, 1347.0
Ref. [23] ^f^	1338.8, 1339.6	1149.0, 1149.8	1145.7, 1146.3	-	-	1341.1, 1342.0

^a^ EA—electron acceptor; ED—electron donor; OP—out-of-phase; IP—in-phase. ^b^ Calculated. ^c^ In argon. ^d^ In nitrogen. ^e^ In krypton. ^f^ In xenon.

**Table 12:** Calculated and experimental bond length and bond angle of the ozone monomer.

	r(OO)/pm	∠OOO/deg
Experimental (ref. [94])	127.2	116.8
MP2 (this work)	128.38	116.62
Difference	1.18	−0.18
CASSCF (this work)	125.51	115.82
Difference	−1.69	−0.98
CCSD/TZ+2Pf (ref. [89])	125.2	117.5
Difference	−1.97	0.7

**Table 13:** Calculated and experimental wavenumbers (cm^−1^) of the ozone monomer.

	ν_1_ (a_1_)	ν_2_ (a_1_)	ν_3_ (b_2_)
Experimental (ref. [91])	1135	716	1089
MP2 (this work)	1157.9	741.5	2244.3
Ratio	1.02	1.04	2.06
CASSCF (this work)	1092.3	770.2	1358.4
Ratio	0.96	1.08	1.25
CCSD/TZ+2Pf (ref. [89])	1280	758	1261
Ratio	1.13	1.06	1.16

## Data Availability

The data presented in this study are available from the author and in the Supplementary Material.

## Supplementary Materials

The following are available online, Figures S1–S5 and Tables S1–S5: titles as indicated in Table of Contents.

## Sample Availability

No physical samples of the compounds are available.
