# Supplementary material for: The Structures, Molecular Orbital Properties and Vibrational Spectra of the Homo- and Heterodimers of Sulphur Dioxide and Ozone. An Ab Initio Study
Source: Molecules. 2021 Jan 25;26(3):626. doi: 10.3390/molecules26030626 (PMC7865749; doi:10.3390/molecules26030626)
Supplement: Supplementary file 1 [file molecules-26-00626-s001.pdf]

Supplementary Material

The Structures, Molecular Orbital Properties and Vibrational Spectra of the Homo- and Heterodimers of Sulphur Dioxide and Ozone. An *ab initio* Study

Thomas A. Ford

Table of Contents

Table S1. Properties of the valence molecular orbitals of sulphur dioxide dimer 5.

Table S2. Properties of the valence molecular orbitals of sulphur dioxide dimer 2.

Table S3. Properties of the valence molecular orbitals of ozone dimer 2.

Table S4. Properties of the valence molecular orbitals of sulphur dioxide-ozone complex 2a. All orbitals are of *a* symmetry.

Table S5. Properties of the valence molecular orbitals of sulphur dioxide-ozone complex 5b.

Figure S1. Valence molecular orbitals of sulphur dioxide dimer 5.

Figure S2. Valence molecular orbitals of sulphur dioxide dimer 2.

Figure S3. Valence molecular orbitals of ozone dimer 2.

Figure S4. Valence molecular orbitals of sulphur dioxide-ozone complex 2a.

Figure S5. Valence molecular orbitals of sulphur dioxide-ozone complex 5b.

Table S1. Properties of the valence molecular orbitals of sulphur dioxide dimer 5.

No.	Symmetry	Energy/*H*	Approximate description [a,b,c]
1-14			core
15	a′	-1.49308	σ(OSO) (EA)
16	a′	-1.48726	σ(OSO) (ED)
17	a″	-1.39319	σ(OSO) (EA)
18	a′	-1.38860	σ(OSO) (ED)
19	a′	-0.88456	lp(S) (EA)
20	a′	-0.87809	lp(S) (ED)
21	a″	-0.70026	lp(O) (EA)
22	a′	-0.69666	σ(S1…O5,O6)
23	a′	-0.69512	σ(S4…O2)
24	a′	-0.68779	lp(O) (ED)
25	a′	-0.65532	π(OSO) (EA)
26	a″	-0.65442	π(OSO) (ED)
27	a″	-0.54556	π(nb)(OSO) (EA)
28	a′	-0.54157	lp(O) (ED)
29	a″	-0.52401	lp(O) (EA)
30	a″	-0.51163	π(nb)(OSO) (ED)
31	a′	-0.50408	n(O) (EA)
32 (HOMO)	a′	-0.49575	lp(O) (ED)
33 (LUMO)	a′	-0.00765	π*(OSO) (EA)
34	a″	-0.00230	π*(OSO) (ED)
35	a′	0.05754	σ*(OSO) (EA)
36	a′	0.06414	σ*(OSO) (ED)
37	a″	0.06859	σ*(OSO) (EA)
38	a′	0.07064	σ*(OSO) (ED)

[a] See Figure 1 for numbering of atoms.

[b] EA – electron acceptor; ED – electron donor.

[c] lp – lone pair; nb – non-bonding.

Table S2. Properties of the valence molecular orbitals of sulphur dioxide dimer 2.

No.	Symmetry	Energy/*H*	Approximate description [a,b]
1-14			core
15	a_g	-1.48846	σ(O3S1O5) + σ(O4S2O6)
16	a_u	-1.48535	σ(O3S1O5) - σ(O4S2O6)
17	a_g	-1.38795	σ(O3S1O5) + σ(O4S2O6)
18	a_u	-1.38760	σ(O3S1O5) - σ(O4S2O6)
19	a_u	-0.88028	lp(S1) – lp(S2)
20	a_g	-0.87636	lp(S1) + lp(S2)
21	a_u	-0.69560	lp(O3S1O5) - lp(O4S2O6)
22	a_g	-0.69536	lp(O3S1O5) + lp(O4S2O6)
23	a_u	-0.68715	lp(O3S1O5) - lp(O4S2O6)
24	a_g	-0.68435	lp(O3S1O5) + lp(O4S2O6)
25	a_g	-0.66656	π(O3S1O5) + π(O4S2O6)
26	a_u	-0.64081	π(O3S1O5) - π(O4S2O6)
27	a_g	-0.54082	π(nb)(O3S1O5) + π(nb)(O4S2O6)
28	a_u	-0.53946	π(nb)(O3S1O5) - π(nb)(O4S2O6)
29	a_g	-0.51556	σ(S1…O4) + σ(S2…O3)
30	a_u	-0.51463	σ(S1…O4) - σ(S2…O3)
31	a_g	-0.49962	lp(O3S1O5) + lp(O4S2O6)
32 (HOMO)	a_u	-0.49343	lp(O3S1O5) - lpO4S2O6)
33 (LUMO)	a_u	-0.00908	π*(O3S1O5) – π*(O4S2O6)
34	a_g	0.00368	π*(O3S1O5) + π*(O4S2O6)
35	a_u	0.05745	σ*(O3S1O5) – σ*(O4S2O6)
36	a_g	0.06510	σ*(O3S1O5) + σ*(O4S2O6)
37	a_u	0.06995	σ*(O3S1O5) – σ*(O4S2O6)
38	a_u	0.07260	σ*(O3S1O5) + σ*(O4S2O6)

[a] See Figure 1 for numbering of atoms.

[b] lp – lone pair; nb – non-bonding.

Table S3. Properties of the valence molecular orbitals of ozone dimer 2.

No.	Symmetry	Energy/*H*	Approximate description [a,b]
1-6			core
7	a_g	-1.73863	σ(O3O1O5) + σ(O4O2O6)
8	a_u	-1.73579	σ(O3O1O5) - σ(O4O2O6)
9	a_u	-1.42452	σ(O3O1O5) - σ(O4O2O6)
10	a_g	-1.42125	σ(O3O1O5) + σ(O4O2O6)
11	a_g	-1.10253	σ(O1…O4) + σ(O2…O3)
12	a_u	-1.09149	lp(O3O1O5) - lp(O4O2O6)
13	a_g	-0.82521	lp(O3O1O5) + lp(O4O2O6)
14	a_u	-0.82494	lp(O3O1O5) - lp(O4O2O6)
15	a_u	-0.80047	lp(O3O1O5) - lp(O4O2O6)
16	a_g	-0.79187	lp(O3O1O5) + lp(O4O2O6)
17	a_g	-0.77739	π(O3O1O5) - π(O4O2O6)
18	a_u	-0.76467	π(O3O1O5) + π(O4O2O6)
19	a_g	-0.56449	π(nb)(O3O1O5) - π(nb)(O4O2O6)
20	a_u	-0.56138	π(nb)(O3O1O5) + π(nb)(O4O2O6)
21	a_g	-0.55901	lp(O3O1O5) + lp(O4O2O6)
22	a_u	-0.54704	lp(O3O1O5) - lp(O4O2O6)
23	a_u	-0.49258	σ(O1…O4) - σ(O2…O3)
24 (HOMO)	a_g	-0.47566	lp(O3O1O5) + lp(O4O2O6)
25 (LUMO)	a_g	-0.05937	π*(O3O1O5) – π*(O4O2O6)
26	a_u	-0.03645	π*(O3O1O5) + π*(O4O2O6)
27	a_u	0.09591	σ*(O3O1O5) – σ*(O4O2O6)
28	a_u	0.09690	σ*(O3O1O5) - σ*(O4O2O6)
29	a_g	0.10330	σ*(O3O1O5) + σ*(O4O2O6)
30	a_g	0.11570	σ*(O3O1O5) + σ*(O4O2O6)

[a] See Figure 3 for numbering of atoms.

[b] lp – lone pair; nb – non-bonding.

Table S4. Properties of the valence molecular orbitals of sulphur dioxide-ozone complex 2a. All orbitals are of *a* symmetry

No.	Energy/*H*	Approximate description [a,b]
1-10		core
11	-1.74704	σ(O4O2O6)
12	-1.48367	σ(O3S1O5)
13	-1.43319	σ(O4O2O6)
14	-1.38485	σ(O3S1O5)
15	-1.11011	lp(O4O2O6)
16	-0,87619	lp(O3S1O5)
17	-0.83491	lp(O4O2O6)
18	-0.80607	lp(O4O2O6)
19	-0.78562	π(O4O2O6)
20	-0.69138	π(nb)(O3S1O5)
21	-0.68221	lp(O3S1O5)
22	-0.65120	π(O3S1O5)
23	-0.57690	π(nb)(O4O2O6)
24	-0.56423	lp(O4O2O6)
25	-0.53722	lp(O3S1O5)
26	-0.51410	lp(O3S1O5)
27	-0.49526	σ(S1…O4) + σ(O2…O3)
28 (HOMO)	-0.49118	σ(S1…O4) - σ(O2…O3)
29 (LUMO)	-0.06202	π*(O4O2O6)
30	0.00537	π*(O3S1O5)
31	0.06597	σ*(O3S1O5)
32	0.07245	σ*(O4O2O6)
33	0.07292	σ*(O3S1O5)
34	0.08376	σ*(O4O2O6)

[a] See Figure 5 for numbering of atoms.

[b] lp – lone pair; nb – non-bonding.

Table S5. Properties of the valence molecular orbitals of sulphur dioxide-ozone complex 5b.

No.	Symmetry	Energy/*H*	Approximate description [a,b]
1-10			core
11	a′	-1.74803	σ(O2O1O3)
12	a′	-1.48894	σ(O5S4O6)
13	a′	-1.43315	σ(O2O1O3)
14	a″	-1.38968	σ(O5S4O6)
15	a′	-1.10438	lp(O2O1O3)
16	a′	-0.87975	lp(O5S4O6)
17	a′	-0.83584	lp(O2O1O3)
18	a′	-0.80403	lp(O2O1O3)
19	a″	-0.78086	π(O2O1O3)
20	a″	-0.69598	lp(O5S4O6)
21	a′	-0.68758	lp(O5S4O6)
22	a′	-0.65703	π(O5S4O6)
23	a′	-0.57122	σ(O2…S4)
24	a′	-0.56105	lp(O2O1O3)
25	a″	-0.54172	lp(O5S4O6)
26	a″	-0.51781	π(nb)(O5S4O6)
27	a′	-0.49842	lp(O5S4O6)
28 (HOMO)	a″	-0.49343	π(nb)(O2O1O3)
29 (LUMO)	a″	-0.05678	π*(O2O1O3)
30	a′	-0.00367	π*(O5S4O6)
31	a′	0.06210	σ*(O5S4O6)
32	a″	0.07117	σ*(O2O1O3)
33	a′	0.07118	σ*(O5S4O6)
34	a′	0.08294	σ*(O2O1O3)

[a] See Figure 5 for numbering of atoms.

[b] lp – lone pair; nb – non-bonding.

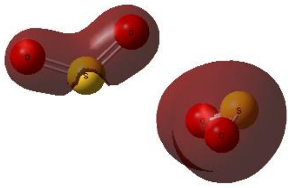

15 (a′)

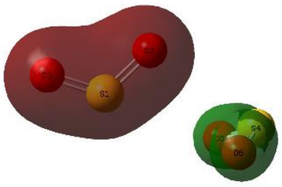

16 (a′)

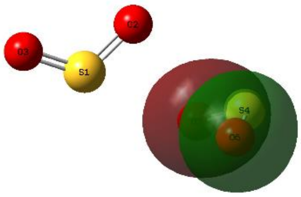

17 (a″)

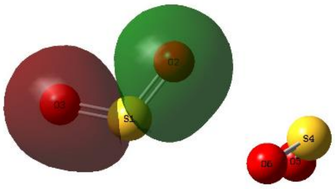

18 (a′)

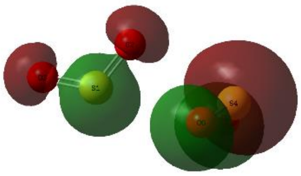

19 (a′)

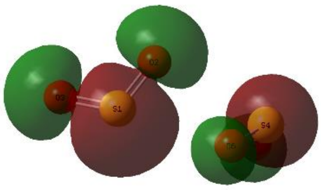

20 (a′)

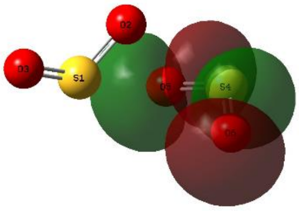

21 (a″)

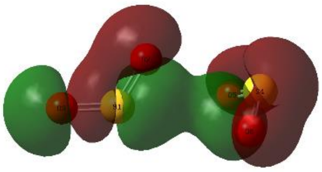

22 (a′)

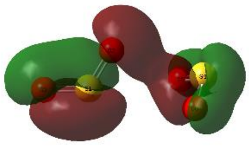

23 (a′)

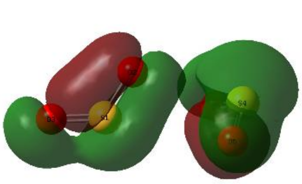

24 (a′)

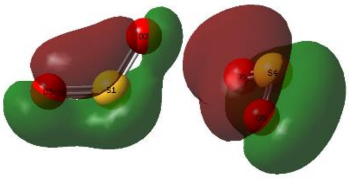

25 (a′)

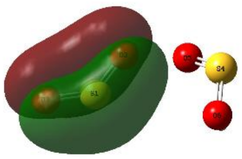

26 (a″)

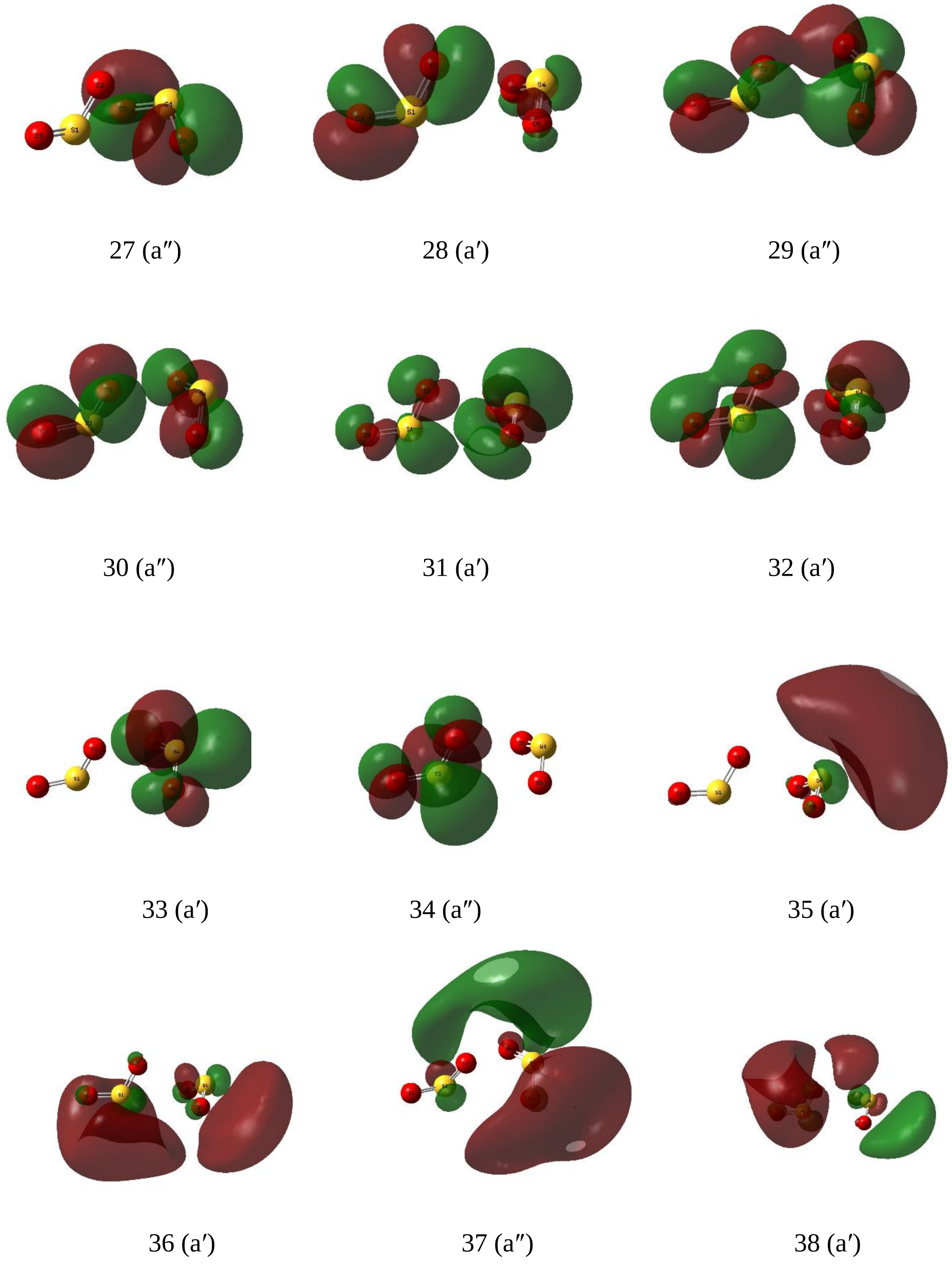

Figure S1. Valence molecular orbitals of sulphur dioxide dimer 5.

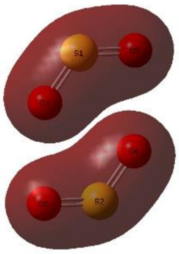

15 (a_g)

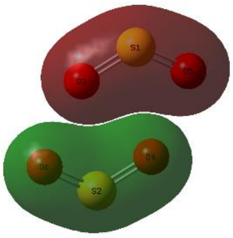

16 (a_u)

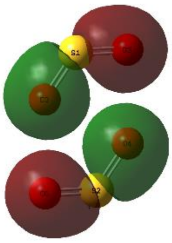

17 (a_g)

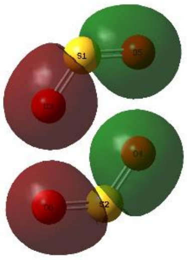

18 (a_u)

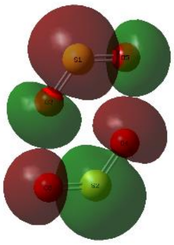

19 (a_u)

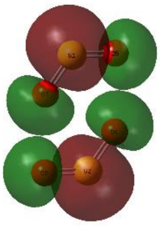

20 (a_g)

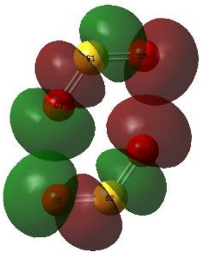

21 (a_u)

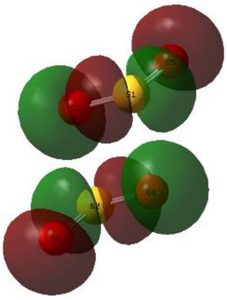

22 (a_g)

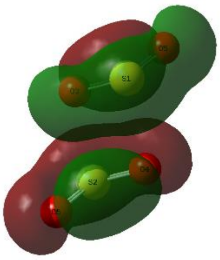

23 (a_u)

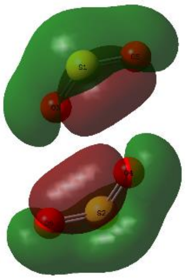

24 (a_g)

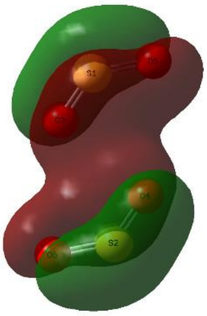

25 (a_g)

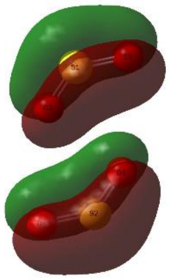

26 (a_u)

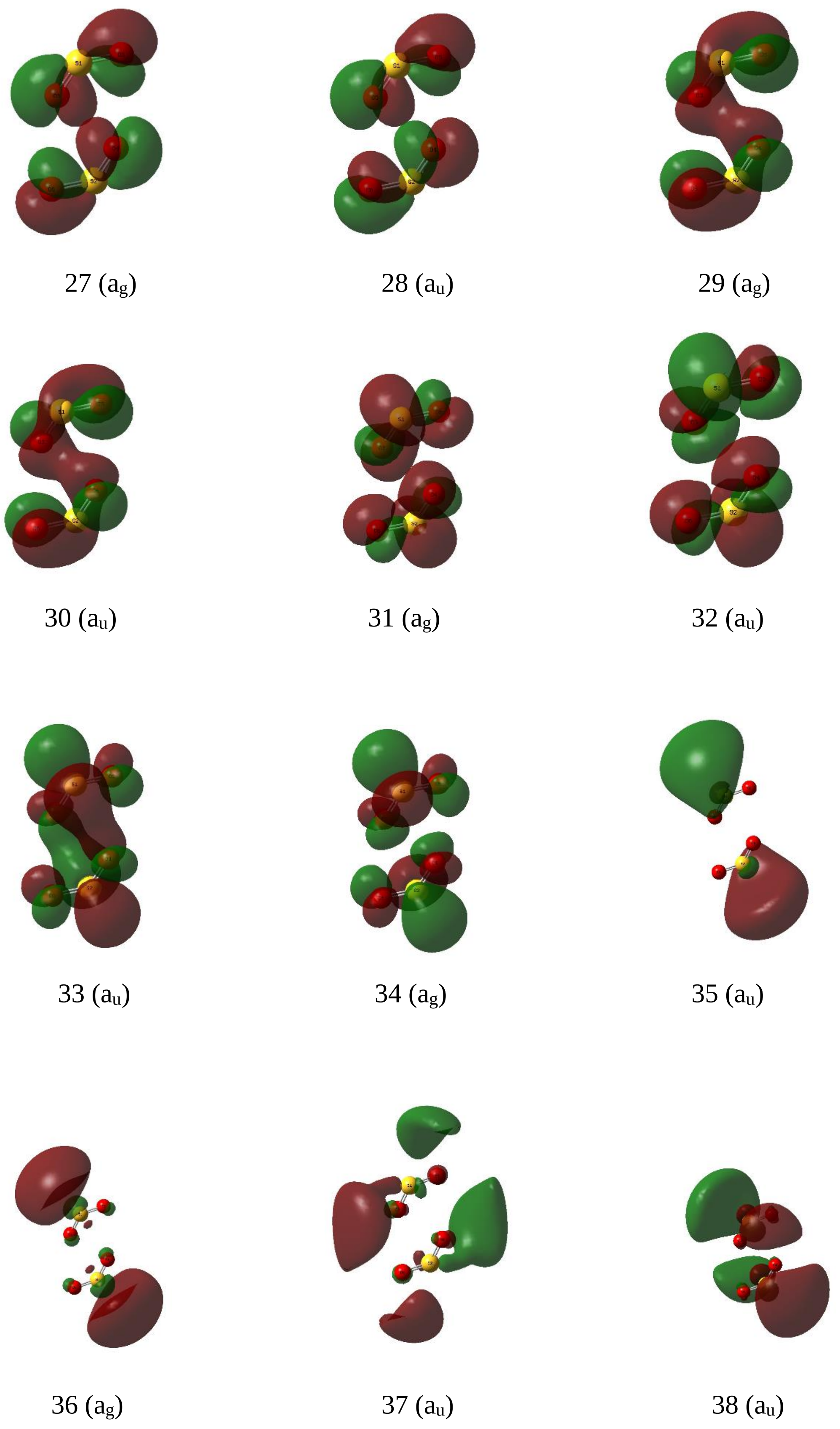

Figure S2. Valence molecular orbitals of sulphur dioxide dimer 2.

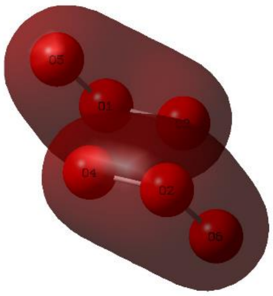

7 (a_g)

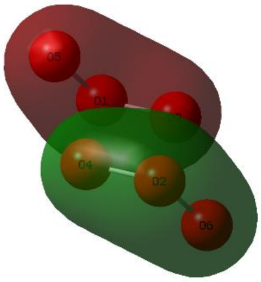

8 (a_u)

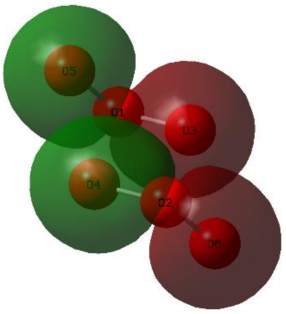

9 (a_u)

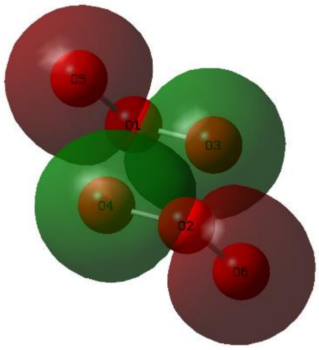

10 (a_g)

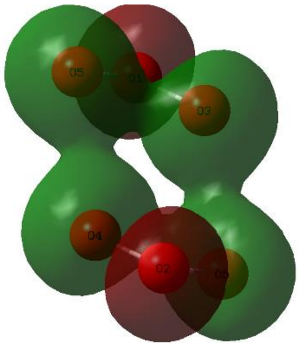

11 (a_g)

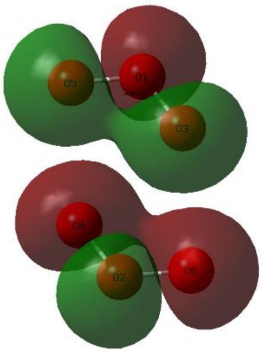

12 (a_u)

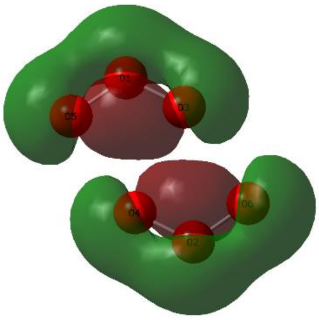

13 (a_g)

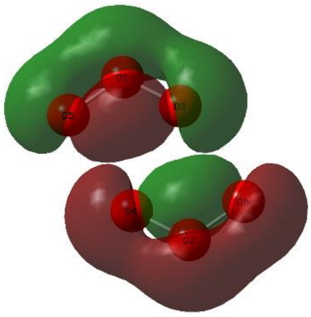

14 (a_u)

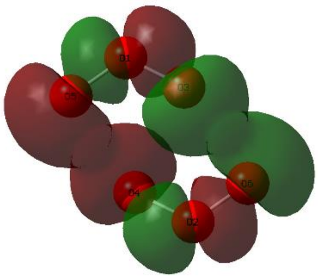

15 (a_u)

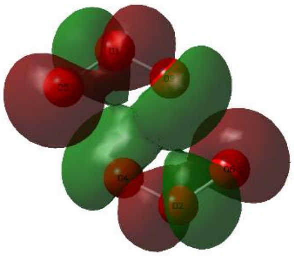

16 (a_g)

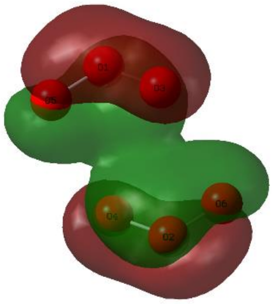

17 (a_g)

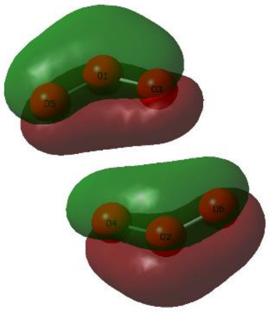

18 (a_u)

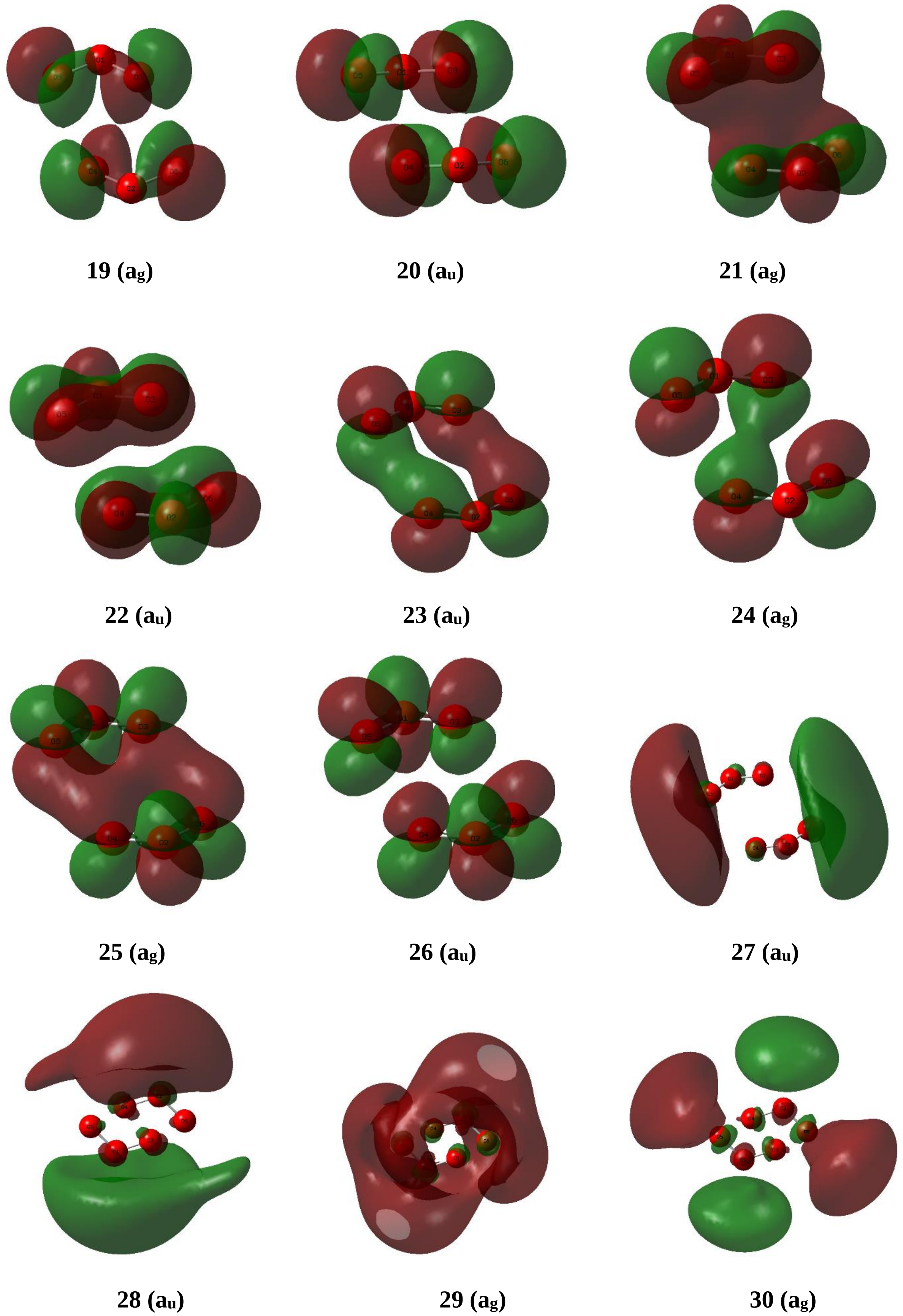

Figure S3. Valence molecular orbitals of ozone dimer 2.

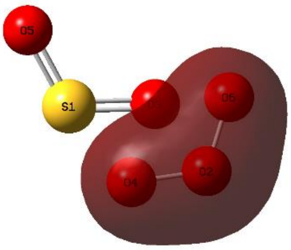

11

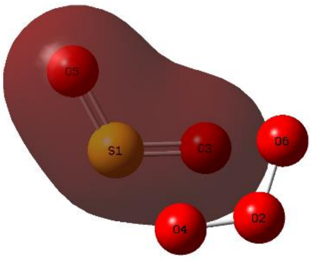

12

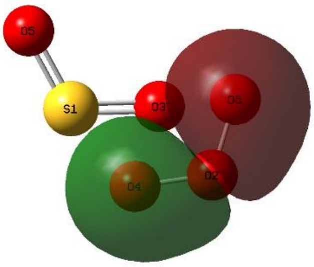

13

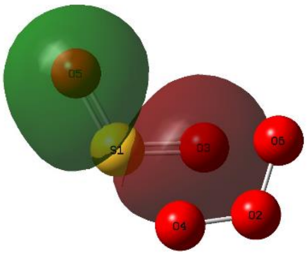

14

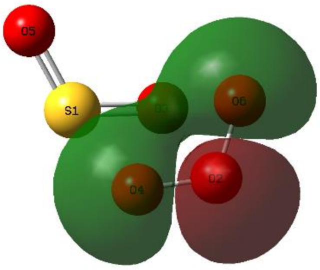

15

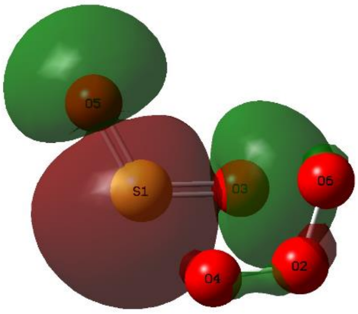

16

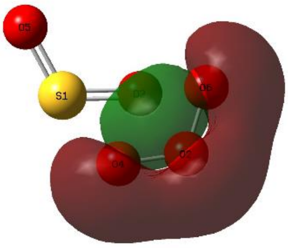

17

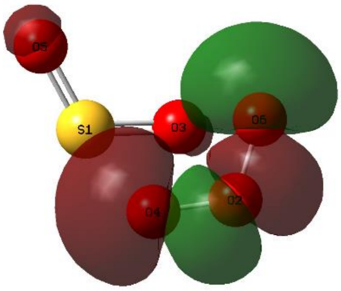

18

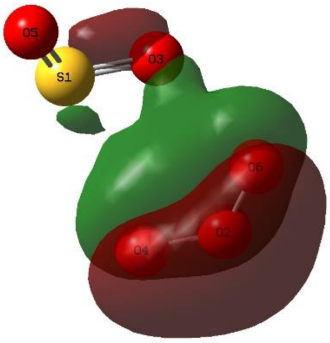

19

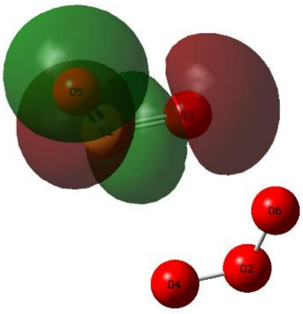

20

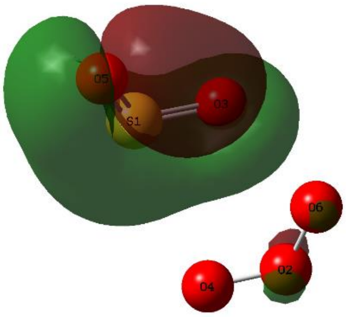

21

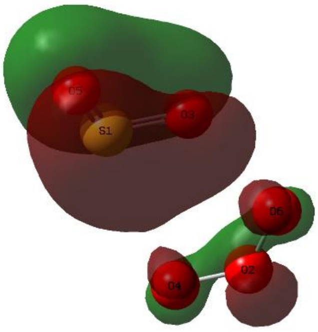

22

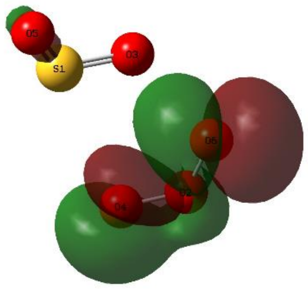

23

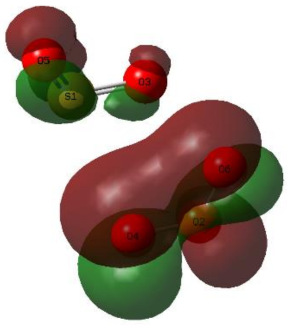

24

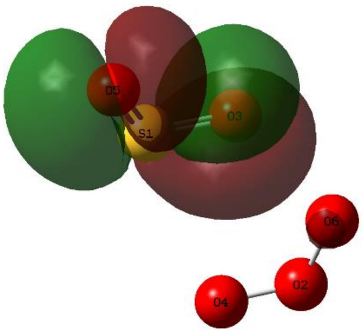

25

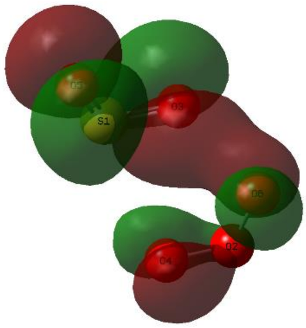

26

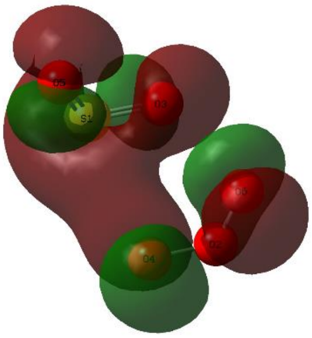

27

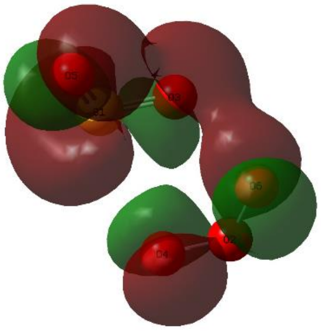

28

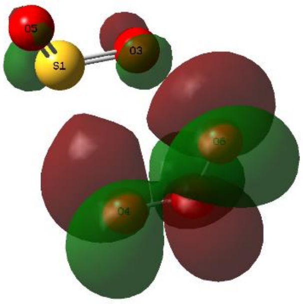

29

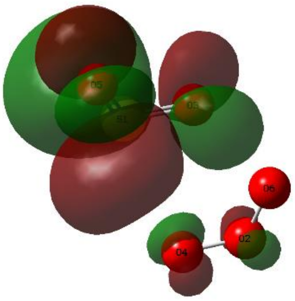

30

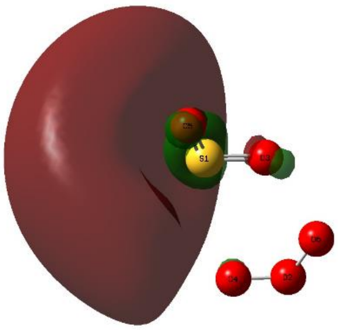

31

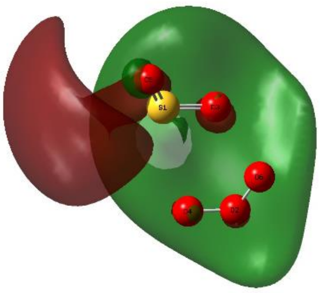

32

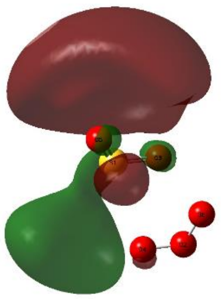

33

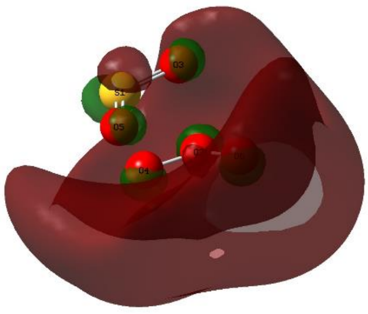

34

Figure S4. Valence molecular orbitals of sulphur dioxide-ozone complex 2a.

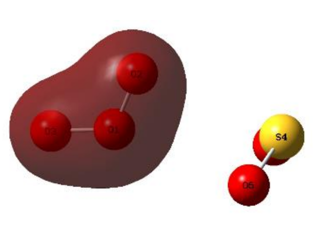

11 (a′)

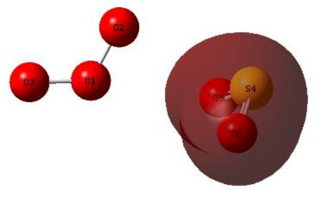

12 (a′)

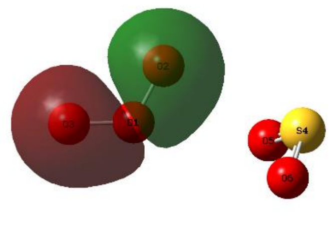

13 (a′)

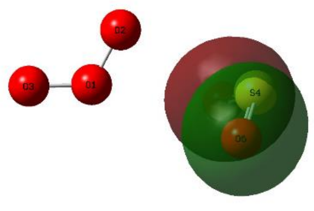

14 (a″)

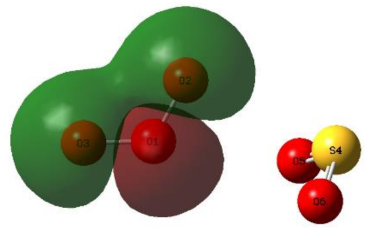

15 (a′)

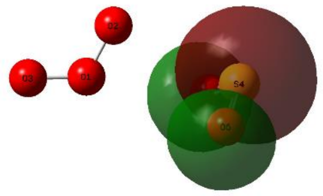

16 (a′)

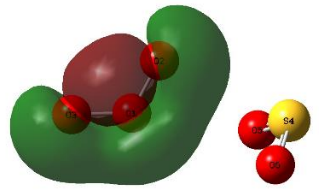

17 (a′)

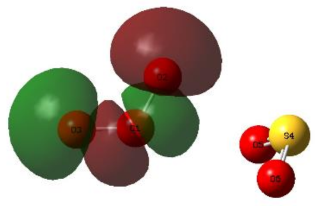

18 (a′)

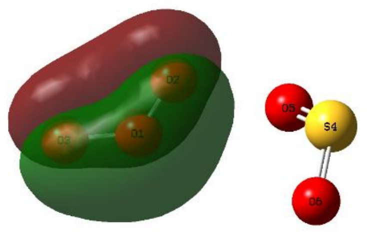

19 (a″)

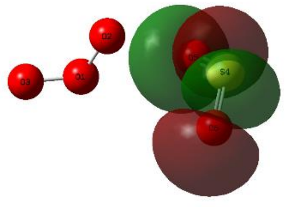

20 (a″)

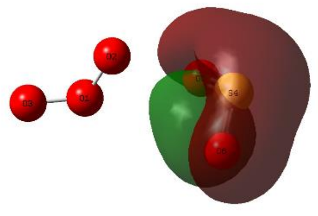

21 (a′)

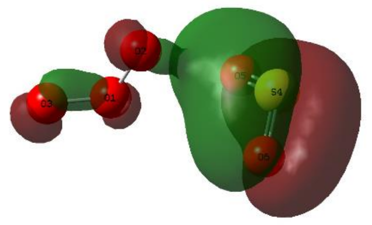

22 (a′)

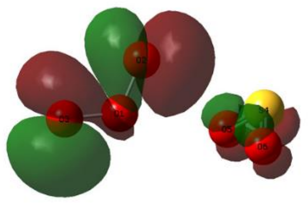

23 (a′)

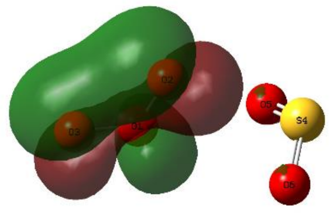

24 (a′)

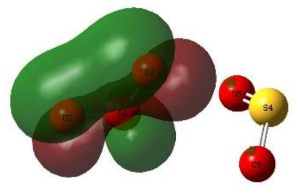

25 (a″)

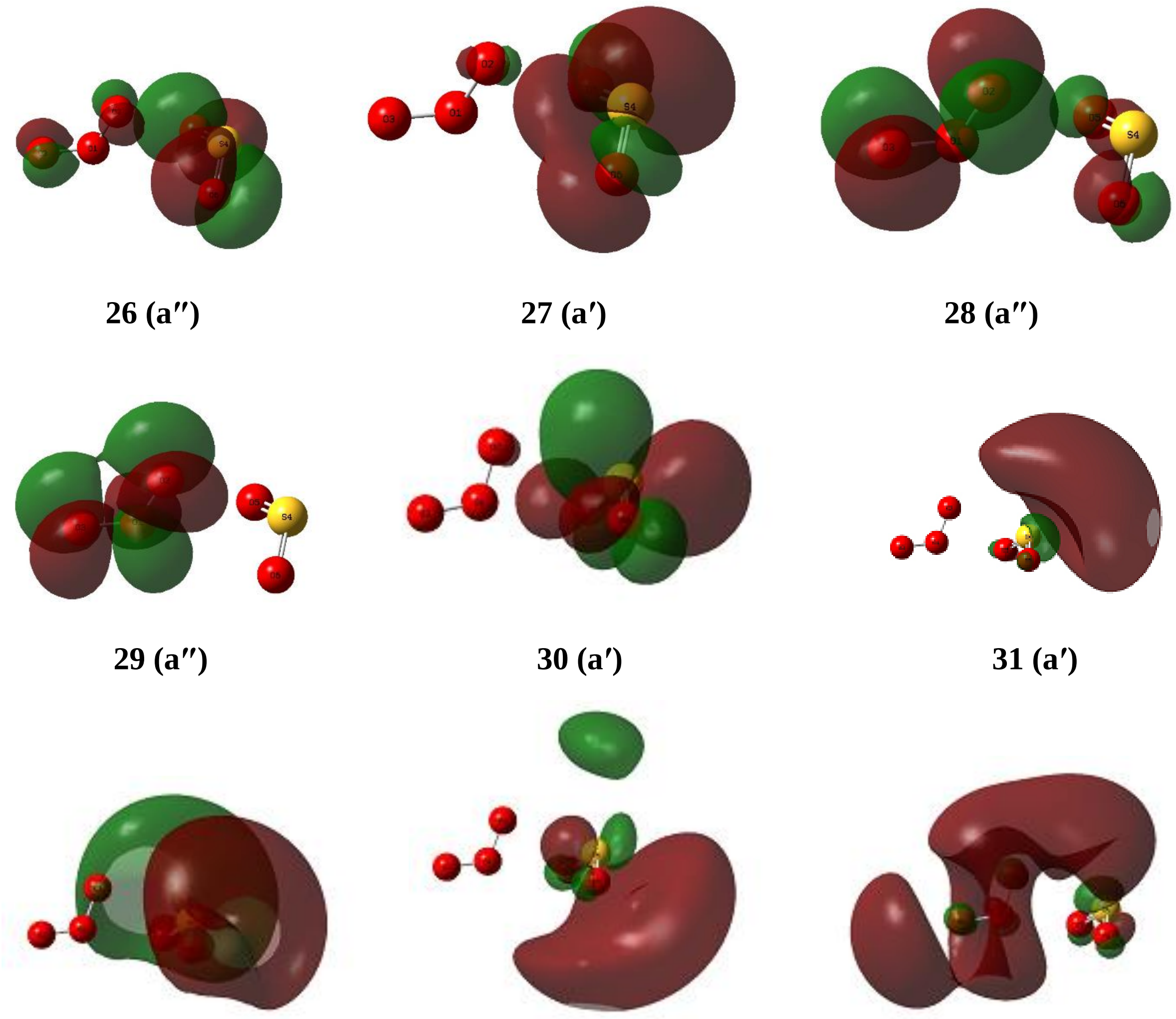

26 (a″) **27 (a′)** **28 (a″)**

29 (a″) **30 (a′)** **31 (a′)**

32 (a″) **33 (a′)** **34 (a′)**

Figure S5. Valence molecular orbitals of sulphur dioxide-ozone complex 5b.
